# Oral Crest Lengthening for Increasing Removable Denture Retention by Means of CO_**2**_ Laser

**DOI:** 10.1155/2014/738643

**Published:** 2014-10-14

**Authors:** Samir Nammour, Elie Gerges, Rima Bou Tayeh, Toni Zeinoun

**Affiliations:** ^1^Department of Dental Sciences, Faculty of Medicine, University of Liege, Liege, Belgium; ^2^Department of Prosthodontic, Faculty of Dentistry, Lebanese University, Beirut, Lebanon; ^3^Department of Oral and Maxillo-Facial Surgery, Faculty of Dentistry, Lebanese University, Beirut, Lebanon

## Abstract

The loss of teeth and their replacement by artificial denture is associated with many problems. The denture needs a certain amount of ridge height to give it retention and a long-term function. Crest lengthening procedures are performed to provide a better anatomic environment and to create proper supporting structures for more stability and retention of the denture. The purpose of our study is to describe and evaluate the effectiveness of CO_2_ laser-assisted surgery in patients treated for crest lengthening (vestibular deepening). There have been various surgical techniques described in order to restore alveolar ridge height by pushing muscles attaching of the jaws. Most of these techniques cause postoperative complications such as edemas, hemorrhage, pain, infection, slow healing, and rebound to initial position. Our clinical study describes the treatment planning and clinical steps for the crest lengthening with the use of CO_2_ laser beam (6–15 Watts in noncontact, energy density range: 84.92–212.31 J/cm^2^, focus, and continuous mode with a focal point diameter of 0.3 mm). At the end of each surgery, dentures were temporarily relined with a soft material. Patients were asked to mandatorily wear their relined denture for a minimum of 4–6 weeks and to remove it for hygienic purposes. At the end of each surgery, the deepest length of the vestibule was measured by the operator. No sutures were made and bloodless wounds healed in second intention without grafts. Results pointed out the efficiency of the procedure using CO_2_ laser. At 8 weeks of post-op, the mean of crest lengthening was stable without rebound. Only a loss of 15% was noticed. To conclude, the use of CO_2_ laser is an effective option for crest lengthening.

## 1. Introduction

The oral rehabilitation of patients after loss of teeth has made significant progress recently. Lack of an adequate residual alveolar ridge and basal seat severely compromises the success of prosthodontic treatment [1]. Vestibuloplasty, ridge augmentation, and different types of implants were used to overcome the problems of flat alveolar ridge [2]. A way of increasing the stability of the prosthesis in this circumstance is to deepen the vestibule [3]; it generally involves increasing by deepening of vestibule without any addition of bone [1]. In the preimplant era, vestibuloplasty was applied to deepen the vestibule with the aim of lining the vestibular trough with functional mucosa in order to form a valve-type margin [4].

Various surgical techniques for vestibular lengthening and vestibuloplasty have been described and advocated. Commonly used in practice, they have the drawback of being associated with a loss of the gained alveolar ridge height of 50% as a result of scar contraction. The patient has to endure pain due to the open wound surfaces and is limited in terms of food intake. In addition, patients often have to reattend the dental practice because they develop pressure sores owing to scar contraction. In the worst-case scenario, the relined denture was not worn by the patient, resulting in conditions similar to the pretreatment situation [5]. The laser technology enables practitioners to achieve a more sustainable result, causing minimal pain to the patient, without the disadvantages of the conventional surgery. The aim of our study is to describe a new method for oral crest lengthening and to evaluate its efficiency and stability.

## 2. Patients and Methods

### 2.1. Patients

Sixty-nine healthy edentulous patients (41 females and 28 males) who needed a crest lengthening in order to improve the stability and retention of their removable dentures were included in this study. The age range of patients was 61–82 years with an average age of 68 years.

Exclusion and inclusion criteria: only patients who had enough distance between the bottom of the vestibule before surgery and nasal spine and with the zygomatic process were reported in this study.

**Figure 1:**
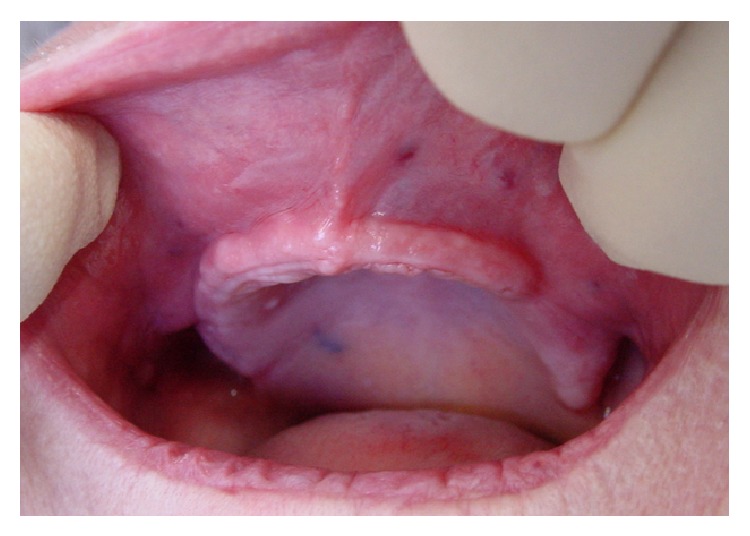
View of the maxilla crest showing a reduced length.

All selected patients were healthy. High-risk cases were excluded because of the absence of suturing of surgical sites at the end of surgeries. According to the declaration of Helsinki and ethic committee recommendations, the decisions for surgery were made after informing patients about the different steps of each surgery, risks, and expected postoperative discomforts. Each surgery was performed only after receiving signed consent of the patient. Only maxilla was included for surgeries (Figure 1) to avoid the risk of trauma of the chin nerves at the mandible. Clinical examination by palpation and the results of X-ray CT scan generating a three-dimensional image of maxilla bone allowed us to make a decision for the surgical option for patients. Only cases for which we were able to perform a vestibular lengthening greater than 10 mm were admitted for this study. No medications were prescribed before surgeries. Due to the financial situation of the patients and the difficulty to afford the cost of prosthetic implant treatments, a surgery by using carbon dioxide laser (CO_2_) was performed.

### 2.2. Laser

CO_2_ laser beam (Smart US20 D Laser 10 600 nm, High Tech Laser, Herzele, Belgium) was used at the following setting: 6–15 Watts, energy density range: 85–212 J/cm^2^, continuous, noncontact, and focus mode, and with a focal point diameter of 0.3 mm.

### 2.3. Surgical Procedure

**Figure 2:**
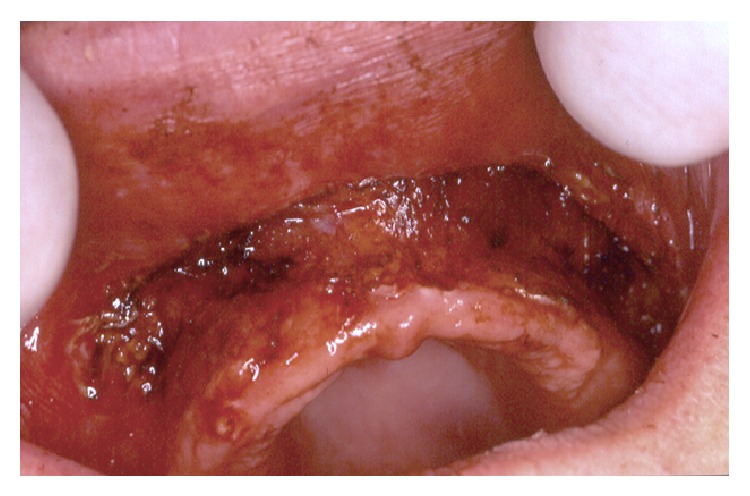
View of the operated site during surgery. The site is bloodless. Neither suture nor graft was necessary.

**Figure 3:**
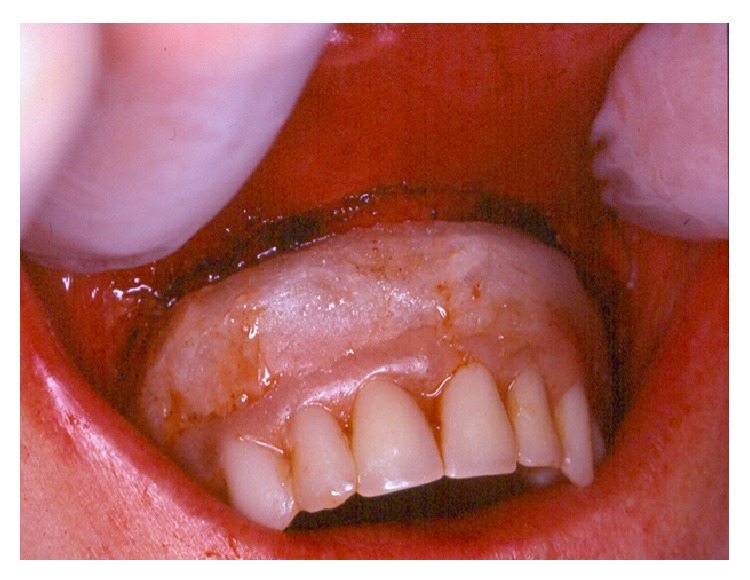
The removable denture was temporarily relined with a soft acrylic material until the bottom of the vestibular deepening. Patients were advised to keep the denture in mouth during the 6 weeks of post-op.

We used a CO_2_ laser machine (10.6 *μ*m) and all treatments were performed in one session. All reported patients were healthy without high-risk cases. Before each surgery, a local anesthesia (articaine with vasoconstrictor) was made. All medical staff wore specified glasses to protect their eyes against CO_2_ laser beam. Patient wore protective eye glasses, or wet and sterile compressive gauzes were applied over the patient's eyes. Surgical procedures were as follows. The incision by CO_2_ laser was always started from the border of attached mucosa of the crest. The incision progressed by leaving 1 mm of soft tissues covering bone surfaces of the maxilla (Figure 2). The incision was carried out with a strict respect of the anatomy of the surface of the maxilla by keeping a parallel trajectory of incisions to the bone surfaces. The incisions for crest lengthening were always stopped 2 mm before the bone surface of the bottom of the vestibule (zygomatic process or nasal spine) (Figure 2) in order to avoid any future discomfort caused by an intensive contact between the edge of the prosthesis and the bone of the vestibule. Sometimes, the incised zone was made up of a very large area from one tuberosity to the other. At the end of surgeries, all old dentures were relined in mouth by a soft acrylic material (Silagum, automix comfort soft relining, DMG inc., Hamburg, Germany) (Figure 3). Patients were asked to mandatorily wear their temporary relined denture for a minimum of 6 weeks after surgery. We allowed patients to remove dentures only for the time of cleaning or for hygienic purposes. At the end of each surgery, the deepest length of the vestibule was measured by means of digital calipers with a precision of 0.01 mm (Caliper digital IP 67 300 mm, Helios-Preisser company, Gammertingen, Germany). No sutures were made and the bloodless wounds healed in second intention without grafts. The aim of the surgery is to remove mussels' attachments in order to increase crest length. The defocus irradiation mode was only used to provoke the coagulation of bleeding areas. The tissue carbonization was gently removed from the operated tissue surfaces. The wound was left without any make-up and without wound dressing.

Postsurgical medication was prescribed: adapted antibiotics with respect to patient allergy risks, nonsteroid anti-inflammatory, analgesics, and a mouthwash solution (chlorhexidine 0.2%).

### 2.4. Follow-Up

Patients were recalled after 2, 4, 6, 8, and 10 weeks. The control concerned the evaluation of healed tissue and the measurement of the deepest vestibular length.

The percentage of success for each surgery has been calculated as follows.

Vestibule length after 2, 6, and 10 weeks divided by the initial vestibule length at the end of surgery = percentage of maintained vestibular lengthening.

ANOVA paired statistical test with Bartlett's test for equal variances (Bartlett's statistic corrected and a posttest: Newman-Keuls multiple comparison test) were used to compare results using the software Graph pad Prism (GraphPad Software, Inc., San Diego, California, USA).

## 3. Results

**Figure 4:**
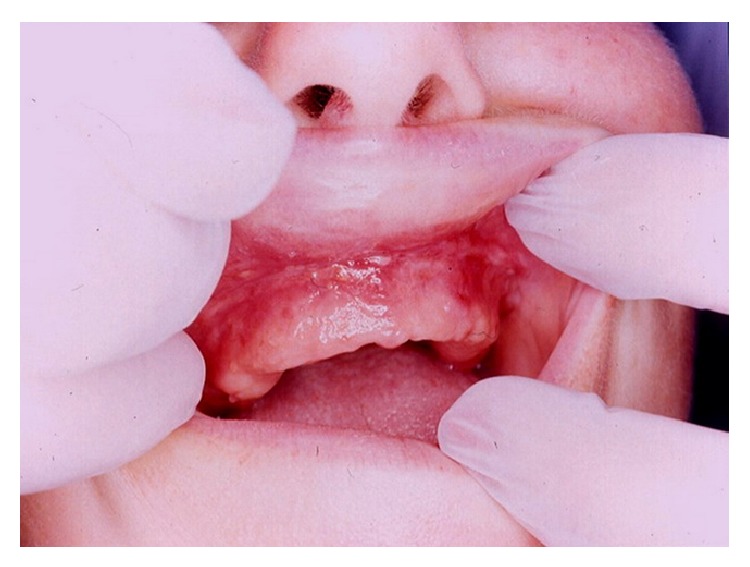
View of the healed site after 4 weeks post-op. The vestibular lengthening is stable. The healing of the crest was satisfactory.

Pain, postoperative edema, and swelling were always present during the postoperative period. In some cases, the complete healing of wounds with a normal tissular aspect was reached after 4 weeks of post-op (Figure 4). During the healing period, the wounds appeared recovered by a layer of fibrin with a white or grey aspect. Despite the healed aspect of the operated area (between 2 and 4 weeks), patients were recommended to wait a minimum of 10 weeks before starting performing a new removable denture or a permanent relining. All operated areas showed a good quality of healing without scar formation.

**Figure 5:**
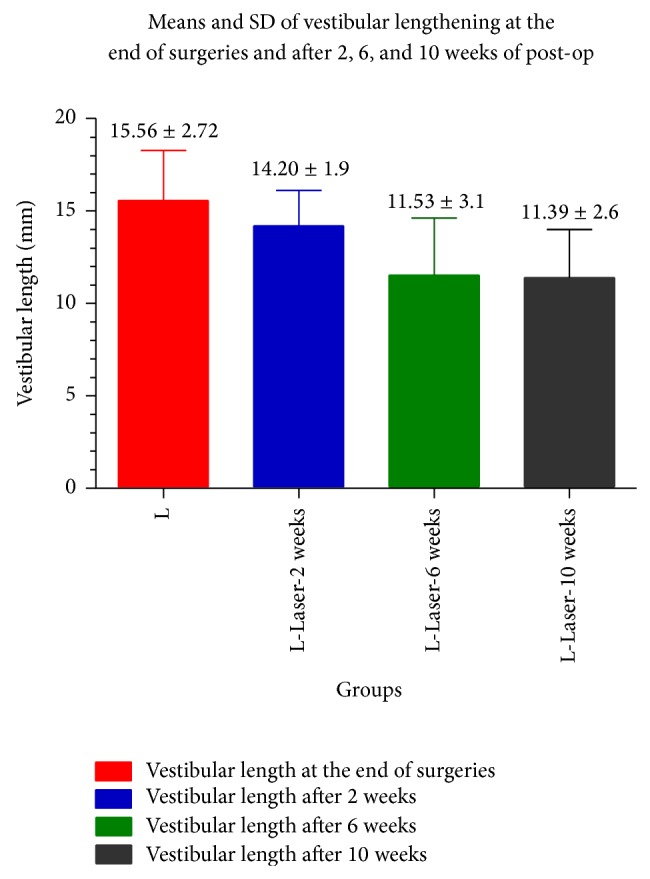
The means and standard deviations of vestibular lengths at the end of surgeries, after 2, 6, and 10 weeks post-op were 15.56 ± 2.72 mm (initial lengths), 14.20 ± 1.9 mm (after 2 weeks of post-op), 11.53 ± 3.1 mm (after 6 weeks), and 11.39 ± 2.6 mm at 10 weeks of post-op. The difference between means of vestibular lengthening in all groups is significantly different except between means of vestibular lengthening after 6 and 10 weeks of post-op. The loss in the initial vestibular deepening continues to increase significantly until 6 weeks post-op and it becomes statically stable.

The means and standard deviations of vestibular lengths at the end of surgeries, after 2, 6, and 10 weeks of post-op, were 15.56 ± 2.72 mm (initial lengths), 14.20 ± 1.9 mm (after 2 weeks of post-op), 11.53 ± 3.1 mm (after 6 weeks), and 11.39 ± 2.6 mm (at 10 weeks of post-op) (Figure 5). The difference between means of vestibular lengthening in all groups is significantly different except between means of vestibular lengthening after 6 and 10 weeks. The loss of the initial vestibular lengthening increased continuously and significantly during the first 6 weeks of post-op and then became statically stable (*P* value <0.0001, *P* value: 0.0012, *R* square: 0.3189, ANOVA test with Bartlett's test for equal variances, Bartlett's statistic corrected with a posttest, and Newman-Keuls multiple comparison test) (Table 1).

The rebound between initial vestibular lengths at the end of surgeries and those at 2, 6, and 8 weeks of post-op may be explained by the tissue remodeling during mussels reattachment.

Failures were reported for three male patients because they could not keep their relined denture in mouth during the postsurgical period (Table 2).

## 4. Discussion

When alveolar ridge resorption occurs in the edentulous mandible, the surface of the attached mucosa on the ridge decreases [6]. In this situation, the connection of the mucosa and muscles near the seat of the complete denture plays an important role in prosthesis retention and stability. It has been suggested that expansion of the denture-bearing area by means of a vestibuloplasty would reduce denture load per square unit of supporting bone and thus reduce the bone resorption caused by transfer of occlusal forces [7].

Many procedures for vestibuloplasty were proposed to overcome the problems of flat alveolar ridge [2]. Vestibular lengthening as a way of increasing the stability of the prosthesis was proposed to deepen the vestibule [3]. This generally involves increasing by deepening of vestibule without any addition of bone [1].

Various surgical techniques for vestibular lengthening and vestibuloplasty have been described and advocated. They have the drawback of being associated with a loss of the gained alveolar ridge height of 50% as result of scar contraction [5]. Previous procedures for vestibuloplasty and crest lengthening proposed the use of grafts (mainly skin graft) [3, 6, 7] or other materials to cover and protect the wound during the healing period. Laser technology enables practitioners to achieve oral surgeries in a bloodless field and without any need for suturing or graft in healthy patients [8–11]. Many authors proposed the use of CO_2_ laser beam for the surgery of vestibular lengthening. The proposed procedures were able to manage a partial and limited vestibular lengthening [12–15]. In our study, we developed a new procedure able to treat a large area of the crest or to manage a global and total vestibular lengthening of the maxilla. Moreover, our surgical protocol pointed out the advantages of using laser beam that can be resumed as follows: an easy and quick procedure, a bloodless surgery, no need for suturing or for graft techniques, and a good quality of healed tissue.

Additionally, the use of CO_2_ laser beam reduces and minimizes dramatically scar formation due to the sparse presence of myofibroblasts in the lased wound. It has been shown that the number of myofibroblasts in CO_2_ laser wounds is three times less than that found in scalpel wounds [16–18]. The result is an appreciated quality of healing in lased tissues without scar formation. This added value of our use of CO_2_ laser for the management of vestibuloplasty resolves the reported problems in conventional procedures (scalpel use): scars in healed tissue and the 50% of rebound.

Moreover, it has been reported that healed mucosa is enriched in collagen induced by laser beam in mast cells and myofibroblasts. The enrichment of lased mucosa by collagen is helpful and allows healed gingival crest to have more resistance to stress caused by dentures [19].

## 5. Conclusion

CO_2_ laser with *λ* = 10.6 *μ*m was effective in crest lengthening procedures and presented some advantages: quick and simple method of applying without any need for suturing or graft techniques. In this way, CO_2_ laser can be reported to be an effective option in improving vestibuloplasty.

## Figures and Tables

**Table 1:** Results of statistical analysis for all groups are shown. Means are statistically different except for means of vestibular lengthening results at 6 and 10 weeks.

ANOVA test				
*P* value	<0.0001			
*P* value summary	∗∗∗			
Are means significantly different? (*P* < 0.05)	Yes			
Number of groups	4			
*F*	42,44			
*R* square	0,3189			

Newman-Keuls multiple comparison test	Mean difference	*q*	Significant? *P* < 0.05?	Summary

L-Laser-10 weeks vs L	−4.170	13.24	Yes	∗∗∗
L-Laser-10 weeks vs L-Laser-2 weeks	−2.810	8.922	Yes	∗∗∗
L-Laser-10 weeks vs L-Laser-6 weeks	−0.1400	0.4445	No	ns
L-Laser-6 weeks vs L	−4.030	12.80	Yes	∗∗∗
L-Laser-6 weeks vs L-Laser-2 weeks	−2.670	8.477	Yes	∗∗∗
L-Laser-2 weeks vs L	−1.360	4.318	Yes	∗∗

**Table 2:** Sexes and number of succeeded and failed surgeries are shown.

	Number of patients	Number of succeeded surgeries	Number of failed surgeries
Females	41	41	0
Males	28	25	3
